# Parliament and the Profession

**Published:** 1916-04-01

**Authors:** 


### PARLIAMENT AND THE PROFESSION.

### Hospital Arrangements in Mesopotamia.

References to medical matters have been unusually numerous in the House of Commons since our last report, and by far the most important of these have been the various statements regarding the Medical Service in Mesopotamia. On March 22 Mr. Chamberlain, alluding to this subject, frankly said : "I am afraid I have to admit that in my opinion there has been a lamentable breakdown of the hospital arrangements." He went on to say that there had apparently been an abundance of hospital supplies of all kinds at Basra at all times, but he thought, without doubt, there had been a grave and, he was inclined to think, an inexcusable shortage of necessary medical supplies above Basra. He intimated that steps had been taken with the object both of apportioning responsibility for the laches of the past and of so improving the arrangements for the treatment of the sick and wounded as would prevent a repetition of the unhappy experience which had given just cause for complaint. In the meantime he begged the House to keep an open mind on the subject. On the following day Mr. Chamberlain, replying to a question by Lord H. Cavendish, with regard to the reason for the delay or refusal of the authorities in accepting offers made by several societies to send large quantities of supplies to Mesopotamia, said that a very generous offer made by the Joint War Committee of the British Red Cross Society and the Order of St. John of Jerusalem on January 31 was not accepted until February 17 because it had been necessary to consult the Viceroy of India, who in turn also had to make inquiries.

### Measures of Relief.

On March 23, Mr. Tennant supplemented the information given by Mr. Chamberlain on the previous day by stating what were the measures to which the Secretary for India had alluded for improving the medical arrangements in Mesopotamia. He said the arrangements were primarily under the control of the military authorities in India, but the Commander-in-Chief in India had been informed that the Army Council would render him any assistance in medical *personnel* and equipment. All demands had been met without delay; 120 medical officers, including 40 from Egypt, and 205 other ranks, Royal Army Medical Corps, had arrived or were on their way. In addition, one British general hospital of 1,040 beds and one British stationary hospital of 400 beds had been sent from Egypt. Both these units were fully equipped. Four Indian general hospitals of 500 beds each, which were in this country, had also been sent. Two of these were fully equipped and the remaining two consisted of *personnel* only. From France the Lahore Indian stationary hospital of 200 beds and thirty-three medical officers were under orders. A motor-ambulance convoy—fifty cars with complete *personnel*—was nearly ready, and would leave for Mesopotamia early in April.

### R.A.M.C. Officers with Special Qualifications.

The desirability of arranging that doctors with special experience in different branches of medicine and surgery who have joined the Royal Army Medical Corps shall be so employed as to utilise to the best advantage their special qualifications was urged by Sir Philip Magnus, whom Mr. Tennant assured that every effort was made to carry out such a policy. But, added Mr. Tennant, it is sometimes not practicable to use their services exclusively in one special line of practice, as the need for special work is limited.

### Other Matters of Interest.

The Royal Commission on Venereal Diseases.—Mr. Lloyd George informed Colonel Lockwood that the recommendations of the Royal Commission on Venereal Diseases are now under consideration.

A Case of Cerebro-spinal Meningitis at Bisley.—Replying to Mr. Newton, Mr. Tennant stated that during the last few weeks only one case of cerebro-spinal meningitis had occurred in the motor machine-gun section at Bisley.

Allowances to Nurses.—The arrangement by which allowances for "light, fuel, and lodgings" were issued to nurses at the Front has been discontinued. The saving thus effected, stated Mr. Forster in reply to a question, is at the rate of about £180,000 a year.

Mental Hospital Attendants.—In reply to a question the Home Secretary stated that the salaries of attendants in mental hospitals who act as orderlies or attend upon nerve-shaken soldiers are, at the request of the War Office, paid by the visiting committees and are afterwards refunded by the War Office.

Venereal Disease in Egypt.—Replying to Colonel Lockwood, Mr. Tennant stated that in Egypt during the last eight weeks the percentage of admissions to hospital for venereal diseases among the troops had averaged 0.09 per cent. per week, this being equal to an average annual admission ratio of 46.8 per thousand.

Visual Standards for Recruits.—Replying to Colonel Yate, Mr. Tennant stated that recruits for the British Army whose standard of vision does not come up to that required for general service are not rejected, but are utilised where it is thought their services would be most valuable, provided that with glasses they can see to shoot.

Cambridge Military Isolation Hospitals.—Cambridge Town Council has drawn the attention of the War Office to the insufficient guards placed at two military isolation hospitals, whereby patients are enabled to escape and thereby cause damage to the health of the community. According to a statement made by Mr. Tennant, this matter is under consideration by the Army Council.

Part-Time Medical Service.—At the present time there is a considerable number of medical practitioners employed in part-time work at hospitals, or in looking after the health of troops stationed in districts where such practitioners reside. Mr. Tennant added that it is anticipated that this number will be largely increased.

Medical Officers of Mental Hospitals.—Medical officers of mental hospitals, adapted as war hospitals, who have been granted temporary commissions in the Royal Army Medical Corps, receive in most cases the pay of their rank unless that is less than their ordinary salary, in which case they are paid at the higher rate. This arrangement, stated the Home Secretary, will continue as long as the institution remains a war hospital.

Dangers of Aeroplane Varnish.—A further fatal case of dope poisoning was referred to by Mr. Rowlands, the explanation of the Home Office being that the system of ventilation, at the factory where the death occurred, for drawing off the fumes was defective in some respects. Steps were being taken to extend the use by contractors of a dope made under War Office supervision, which contains as little tetrachlorethane as possible.

